# Genetic variation and marker−trait association affect the genomic selection prediction accuracy of soybean protein and oil content

**DOI:** 10.3389/fpls.2022.1064623

**Published:** 2022-12-13

**Authors:** Bo Sun, Rui Guo, Zhi Liu, Xiaolei Shi, Qing Yang, Jiayao Shi, Mengchen Zhang, Chunyan Yang, Shugang Zhao, Jie Zhang, Jianhan He, Jiaoping Zhang, Jianhui Su, Qijian Song, Long Yan

**Affiliations:** ^1^ Institute of Cereal and Oil Crops, Hebei Academy of Agricultural and Forestry Sciences, Shijiazhuang Branch Center of National Center for Soybean Improvement, The Key Laboratory of Crop Genetics and Breeding, Shijiazhuang, China; ^2^ College of Life Sciences, Hebei Agricultural University, Baoding, China; ^3^ State Key Laboratory of Crop Genetics and Germplasm Enhancement, National Center for Soybean Improvement, Nanjing, China; ^4^ Key Laboratory for Biology and Genetic Improvement of Soybean, (General, Ministry of Agriculture), Nanjing Agricultural University, Nanjing, China; ^5^ Agricultural-Regionalization Workstation of Shijiazhuang’s Gaocheng District, Shijiazhuang, China; ^6^ Soybean Genomics and Improvement Laboratory, Agricultural Research Service, United States Department of Agriculture, Beltsville, MD, United States

**Keywords:** soybean, protein content, oil content, GS, prediction accuracy

## Abstract

**Introduction:**

Genomic selection (GS) is a potential breeding approach for soybean improvement.

**Methods:**

In this study, GS was performed on soybean protein and oil content using the Ridge Regression Best Linear Unbiased Predictor (RR-BLUP) based on 1,007 soybean accessions. The SoySNP50K SNP dataset of the accessions was obtained from the USDA-ARS, Beltsville, MD lab, and the protein and oil content of the accessions were obtained from GRIN.

**Results:**

Our results showed that the prediction accuracy of oil content was higher than that of protein content. When the training population size was 100, the prediction accuracies for protein content and oil content were 0.60 and 0.79, respectively. The prediction accuracy increased with the size of the training population. Training populations with similar phenotype or with close genetic relationships to the prediction population exhibited better prediction accuracy. A greatest prediction accuracy for both protein and oil content was observed when approximately 3,000 markers with -log_10_(*P*) greater than 1 were included.

**Discussion:**

This information will help improve GS efficiency and facilitate the application of GS.

## Introduction

Soybean (*Glycine max* L.) is an important crop worldwide (Babu et al., 2016). It accounts for approximately 68% of the world’s protein and 27.7% of the vegetable oil used for human consumption (https://downloads.usda.library.cornell.edu/usda-esmis/files/tx31qh68h/2f75sh11z/7d27b176h/oilseeds). The increase in soybean protein and oil content are important goals for soybean breeding. It has been widely documented that soybean seed composition is not only affected by abiotic and biotic factors but also genetically controlled (Rodrigues et al., 2014). The seed protein and oil content of the same accessions varied in different years or in different environments within the same year (Helms et al., 1990). Marker-assisted selection (MAS) can improve the efficiency of breeding selection and has been used for the selection of soybean salt tolerance (Lee et al., 2004), insect resistance (Zhu et al., 2006) and other agronomic traits (Zhang et al., 2015). However, MAS relies on the degree of marker association with the quantitative trait loci and the amount of genetic effect of the quantitative trait locus (QTL) explained (Moreau et al., 2004). The trait-related loci detected by QTL mapping and genome-wide association studies (GWAS) may not be dominant across different populations, which also reduces the efficiency of MAS (Beavis, 1998; Melchinger et al., 1998; Moreau et al., 1998). In addition, QTL analyses may only capture a portion of the genetic variation contributed to the targeted trait, while many loci with small effects or susceptible to environmental influence go undetected (Maher, 2008). Genomic selection (GS) was different from MAS in that it used a large number of molecular markers to estimate the breeding value or genetic value and predict the performance of candidate individuals without identifying the marker−trait association (Budhlakoti et al., 2022). It could overcome the limitations of MAS in breeding and was an effective method for selecting complex traits.

With the development of high-throughput genotyping techniques and the continuous optimization of statistical models, the application of GS to breeding has become possible. GS was initially used in the breeding of livestock and poultry, such as dairy cows (Pryce and Daetwyler, 2011), pigs (Cleveland and Hickey, 2013), chickens (Calenge et al., 2011), sheep (Duchemin et al., 2012) and other animals. At the beginning of the 21^st^ century, the GS was being explored in many crops . Prediction accuracy is the parameter used to measure the performance of GS (Jarquin et al., 2016; Zhang et al., 2016; Duhnen et al., 2017). Spindel et al. (2015) performed GS on a panel of 363 rice elite breeding lines and found that the predictive abilities for grain yield, plant height and flowering time were 0.31, 0.34, and 0.63, respectively. In maize, the prediction accuracy for the representative subset selection methods, i.e. maximization of connectedness and diversity (MaxCD), partitioning around medoids (PAM) and fast and unique representative subset selection (FURS), was higher than random sampling for flowering time, ear height, and grain yield (Xu et al., 2021). When applying GS to crop breeding, several genetic factors should be considered, including marker density, sample size, the relationship between the training population and test population, population structure, heritability and genetic architecture of target traits, and linkage disequilibrium (LD) between markers and QTLs (Xu et al., 2021). In soybean, Xavier et al. (2016) explored the effect of training population size on prediction accuracy for plant height, days to maturity, number of reproductive nodes, pods per node, number of pods, and grain yield and found that the subset size was an important factor for the improvement of genome-wide prediction. Duhnen et al. (2017) performed GS on soybean yield and seed protein content and compared the genomic prediction accuracy between intersubgroup and intrasubgroup calibration models. The results showed that intra-subgroup calibration was more effective. Ravelombola et al. (2021) explored the effects of population structure on the prediction accuracy of yield-related traits in soybean. The results showed that the yield-related traits could be selected through GS. Qin et al. (2019) conducted GWAS and GS for amino acid concentrations in soybean seeds and showed that the selection efficiency of amino acids based on the markers significantly associated with all 15 amino acids was higher than that based on random markers. There has been little exploration of the predictive accuracy of genomic selection for soybean oil content.

This study aimed to evaluate a number of factors, including the training population sizes, phenotypic and genotypic similarities between training populations and prediction populations and number of markers and marker−trait association, on the accuracy of genome selection of protein and oil content based on 1,007 soybean germplasms genotyped with SoySNP50K BeadChip (Song et al., 2013).

## Materials and methods

### Plant materials and phenotyping

A cultivated soybean panel, Max_IL_0102, consisting of 1,007 soybean germplasms (**Table S7**), was used in this study. The 1,007 soybean germplasms were from the USDA Soybean Germplasm Collection. The entire collection consists of nearly 22,000 accessions, including modern and land race cultivars (*G*. *max*) wild relatives of soybean (*G*. *soja*), and perennial *Glycine* (www.soybase.org). The 1,007 germplasms were originally from China, South Korea, Japan, North Korea, Vietnam, Ukraine, Russia and the United States, and the accessions were maturity groups 0, I, II, III, IV and V. The datasets containing the protein and oil content of the germplasms were downloaded from the GRIN (http://www.ars-grin.gov/npgs/searchgrin.html). According to the GRIN, the field trials of the panels were conducted at Urbana, Illinois. The planting dates were May 4, 2001, and May 27, 2002. The plots were 4 m long with 4 rows 76 cm apart. They were trimmed to 2.4 m after maturity, and the middle two rows were harvested. Protein and oil content in yellow seed soybean accessions were evaluated using the near-infrared reflectance method, and protein and oil content in pigmented or mottled soybean accessions were quantified with the Kjeldahl method and Butt extraction method, respectively. SPSS 25.0 (Shen et al., 2020) was used to calculate descriptive statistics of the phenotypic data, including minimum, maximum, mean, standard deviation, variance, skewness, kurtosis and coefficient of variation.

### Genotyping

The SNP genotypes of 1,007 soybean germplasms were determined using an Illumina Infinium SoySNP50K BeadChip at USDA-ARS, Beltsville, MD, USA. The dataset was obtained from the Soybean Genetics and Improvement Laboratory, USDA-ARS, Beltsville, MD, USA. The chip contained 52,041 SNPs from the euchromatic and heterochromatic regions of the soybean genome. Single nucleotide polymorphism genotyping was conducted on the Illumina platform by following the InfiniumH HD Assay Ultra Protocol (Illumina, Inc.). The SNPs were scored using the GenomeStudio Genotyping Module v1.8.4 (Illumina, Inc.). The SNP data is publicly available at http://www.soybase.org/dlpages/index.php (Song et al., 2015). After eliminating SNPs with a minor allele frequency less than 5%, a total of 42,509 high-quality SNPs were retained for further analysis.

### Genomic selection

GS was conducted using a ridge regression best linear unbiased predictor (RR-BLUP) model (Endelman, 2011) as in the follows.


y = 1nμ + Zu + ϵ

In this equation, y represents the n × 1 dimensional observation vector; 1n denotes the n × 1 dimensional vector with the value of each element 1; μ is the fixed effect; Z represents the n × m design matrix related to the random effect; u is the random effect vector; and ϵ is the vector (n × 1) of independent random residuals. The prediction accuracy was measured by the correlation coefficient between the predicted value and the observed value. The intragroup analysis was repeated 150 times in each case, and the average of the correlation coefficients was used as a measure of the GS prediction accuracy.

### Training population selection

Nine training population sizes ranging from 100 to 900 with an increment of 100 were chosen. In each GS analysis, germplasms were randomly selected to be included in the training population, and the remaining accessions were included in the prediction population.

To evaluate the influence of the phenotypic similarity between the training population and prediction population on prediction accuracy, 1,007 soybean germplasms were sorted according to their phenotypic value and divided into four subpopulations with the same population size. Each subpopulation included approximately 250 soybean germplasms. Genomic prediction analyses between pairwise subpopulations were performed. A fivefold cross-validation scheme was used to assess the prediction accuracy within each subpopulation.

To study the effect of genetic similarity between the training population and prediction population on the prediction efficiency, population structure was determined using STRUCTURE 2.3.4 (Pritchard et al., 2000). A total of 7,244 SNPs with linkage disequilibrium (LD) less than 0.50 to adjacent loci were selected using the program PLINK (version 1.07) (Purcell et al., 2007; Yan et al., 2019) and used to perform the structure analysis. The number of subsets (k) ranged from 2 to 6, while the burn-in time and iterations for each run were both set to 100,000. Two runs were used for each k (Evanno et al., 2005). Then, two subpopulations with the most germplasm were selected for GS analysis, and each subpopulation was used as a training population and a prediction population for mutual prediction. At the same time, 80% of the germplasms in the two subpopulations with the most germplasms were randomly selected as the training population, and the remaining 20% of the germplasm were predicted. The resulting prediction accuracies were then presented in scatterplots. Principal component analysis (PCA) was also used to ensure clear subpopulations.

### Marker selection

To study the effect of marker and trait association on prediction accuracy, the -log_10_(*P*) for each SNP was estimated through GWAS. Genome-wide SNP marker and trait association analyses were performed using a mixed linear model including principal component analysis (PCA) and a kinship matrix (K) as covariates in the analysis. The analysis was performed using the standalone software TASSEL V5.2.9 (Bradbury et al., 2007). The mixed linear model (MLM) considering both population structure and kinship was employed for GWAS using TASSEL software. The MLM was the following:


y = μ + Xα + Pβ + Zu + e

where y is the vector of phenotypic observations; μ is the overall mean; α is the vector of SNP effects; X is the incidence matrix relating the individuals to the fixed marker effects; α and β are the vectors of population structure effects; P is the incidence matrix relating the individuals to the fixed population structure effects; u is the vector of kinship background effects; Z is the incidence matrix relating the individuals to the random group effects; and u and e are the vectors of residual effects (Yu et al., 2006).

Based on the significance level of the association of SNPs with each trait in the GWAS, the SNPs were divided into five sets: set 1 included all the SNPs, and sets 2, 3, 4, and 5 included the SNPs with -log_10_(*P*)>1, -log_10_(*P*)>2, -log_10_(*P*)>3 and -log_10_(*P*)>4, respectively. The same number of markers in the five sets were randomly selected for GS analysis. Fivefold cross-validation was performed. In each cross-validation, 80% of the samples were randomly selected as the training set, and the remaining samples were selected as the validation set.

## Results

### Phenotyping

The protein content ranged from 345 mg/g to 529 mg/g with an average of 434.2 mg/g, the skewness was 0.14, the kurtosis was -0.21, and the coefficient of variation was 6.9%. The oil content ranged from 82 mg/g to 240 mg/g with an average of 174.3 mg/g, the skewness, the kurtosis, and the coefficient of variation was 0.15, -0.09 and 13.4%, respectively 1,007 (**Table 1**).

**Table 1:** Descriptive statistical analysis of the protein and oil content (mg/g) of 1,007 soybean germplasms grown at Urbana, IL, in 2001 and 2002.

Trait	Min	Max	Average	SD	variance		Skewness	Kurtosis		Coefficient of variation (%)
Protein	345	529	434.2	30.0		9.03	0.14		-0.21	6.9
Oil	82	240	174.3	23.4		5.43	0.15		-0.09	13.4

### Effect of training population size on the accuracy of GS

**Figure 1:**
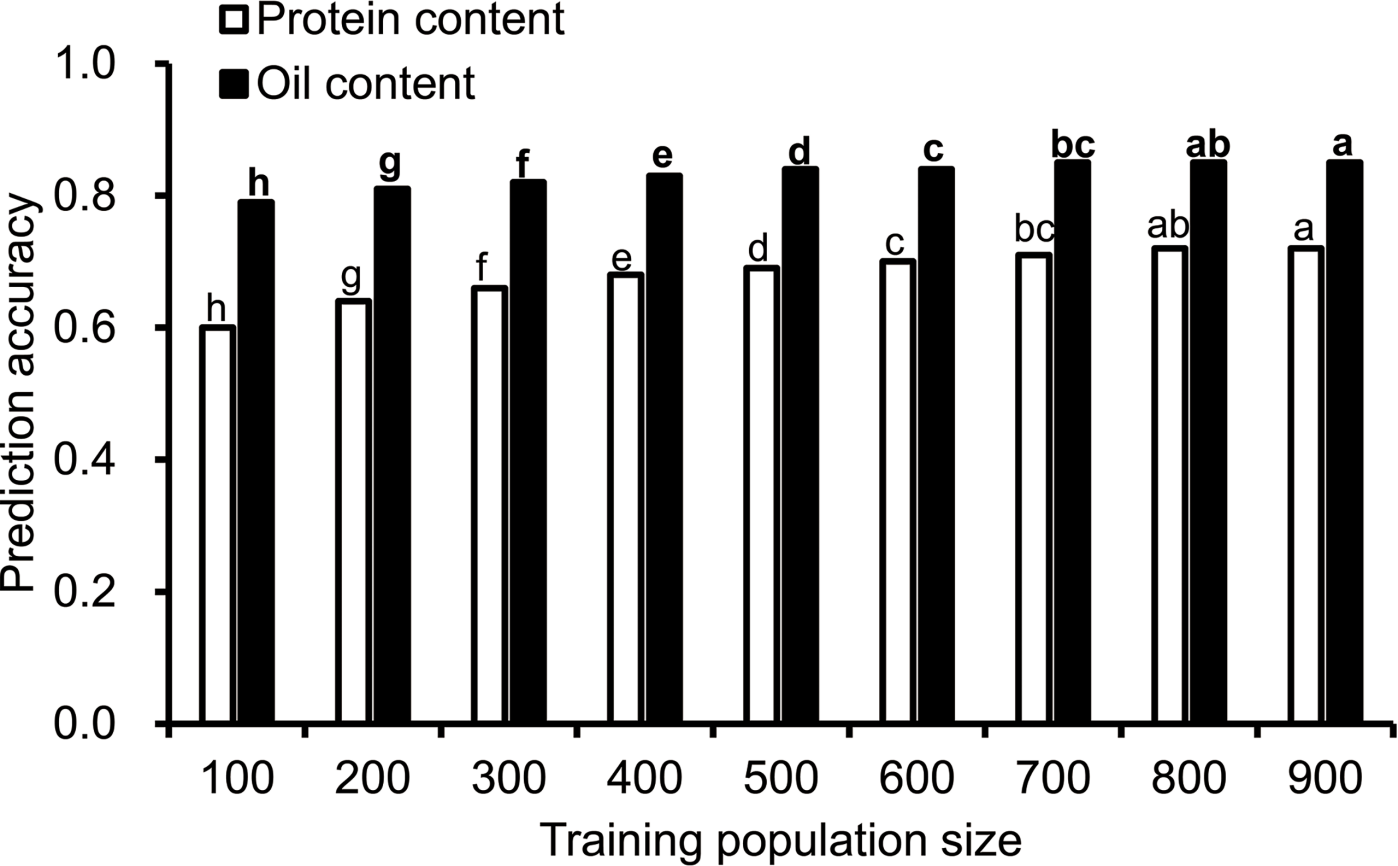
The influence of training population size on prediction accuracy. Cross-validated prediction accuracies of ridge regression best linear unbiased prediction (RR-BLUP) for protein and oil content among 1,007 soybean germplasms grown at Urbana, IL in 2001 and 2002. "a–h" indicates the difference between different columns. The difference between columns marked with different letters was significant (P<0.05), and there was no significant difference between columns marked with the same letter.

To understand the effect of training population size on the prediction accuracy, nine training population sizes ranging from 100 to 900 were chosen. The prediction accuracy for protein and oil content ranged from 0.6 to 0.85, and the prediction accuracy was higher for oil content than protein content (**Figure 1**). The prediction accuracy of protein content was the lowest (0.6) when the training population size was 100, and the accuracy increased with the training population size and reached its maximum value when the training population size was 800. The prediction accuracy for oil content increased from 0.79 to 0.85 when the training population size increased from 100 to 900. It reached a maximum value when the training population size was 800.

### Effect of phenotype similarity between the training population and prediction population on prediction accuracy

**Figure 2:**
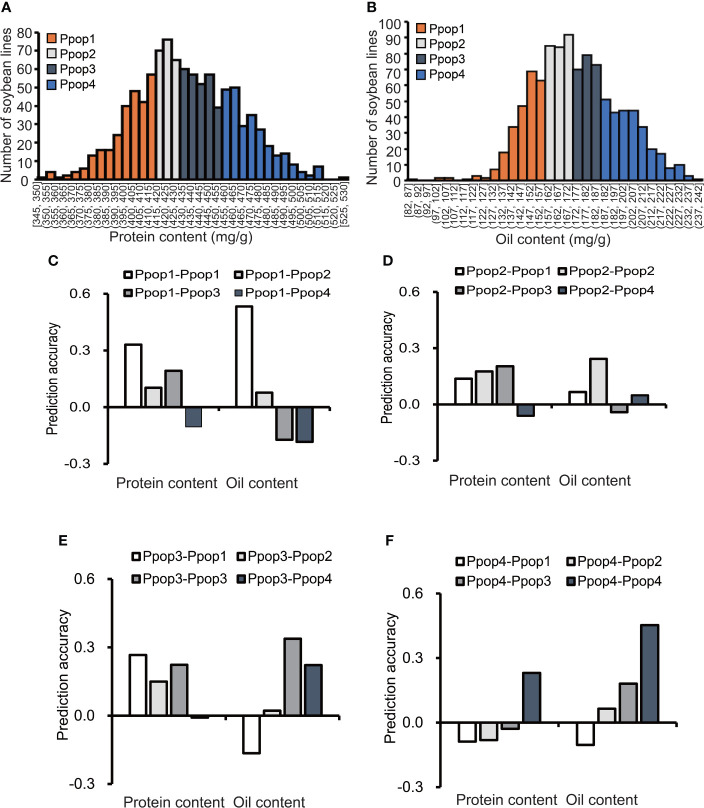
The influence of the phenotypic similarity between the training population and prediction population on prediction accuracy. The prediction accuracies of RR-BLUP for protein and oil content **(C–F)** among soybean germplasms grown at Urbana, IL in 2001 and 2002, when the training population and prediction population were selected based on phenotype **(A, B)**.

According to the protein content distribution, the 1,007 soybean germplasms were divided into 4 subpopulations, each containing 250 accessions (**Table S1**, **Figure 2A**). The range of the protein content (mg/g) for Ppop 1 (phenotypic subpopulation 1), Ppop 2, Ppop 3 and Ppop 4 was 345 to 413, 414 to 432, 432 to 455 and 456 to 529, respectively. When Ppop 1 was used as the training population to predict the protein content of Ppop 1, Ppop 2, Ppop 3, and Ppop 4, the prediction accuracy was 0.33, 0.1, 0.19, and -0.1, respectively. When Ppop 2 was used as the training population to predict the protein content of Ppop 1, Ppop 2, Ppop 3, and Ppop 4, the prediction accuracy was 0.14, 0.18, 0.20, and -0.06, respectively. When Ppop 3 was used as the training population to predict the protein content of Ppop 1, Ppop 2, Ppop 3, and Ppop 4, the prediction accuracy was 0.27, 0.15, 0.22, and -0.01, respectively. When Ppop 4 was used as the training population to predict the protein content of Ppop 1, Ppop 2, Ppop 3, and Ppop 4, the prediction accuracy was -0.09, -0.08, -0.03, and 0.23, respectively (**Figures 2C–F**). When three of the four phenotypic subpopulations were used as the training group to predict the protein content of the remaining subpopulation, the prediction accuracy were 0.19, 0.22, 0.24 and 0.02, respectively (**Table S2**). The 1,007 soybean germplasms were also divided into 4 subpopulations based on their oil content (**Table S1**, **Figure 2B**). The range of the oil content (mg/g) in Ppop 1, Ppop 2, Ppop 3 and Ppop 4 was 82 to 158, 158 to 172, 172 to 189 and 189 to 240, respectively. When Ppop 1 was used as the training population to predict the oil content of Ppop 1, Ppop 2, Ppop 3, and Ppop 4, the prediction accuracy was 0.53, 0.08, -0.17, and -0.18, respectively. When Ppop 2 was used as the training population to predict the oil content of Ppop 1, Ppop 2, Ppop 3, and Ppop 4, the prediction accuracy was 0.07, 0.24, -0.04, and 0.05, respectively. When Ppop 3 was used as the training population to predict the oil content of Ppop 1, Ppop 2, Ppop 3, and Ppop 4, the prediction accuracy was -0.16, 0.02, 0.34, and 0.22, respectively. When Ppop 4 was used as the training population to predict the oil content of Ppop 1, Ppop 2, Ppop 3, and Ppop 4, the prediction accuracy was -0.10, 0.06, 0.18, and 0.45, respectively (**Figures 2C–F**). When three of the four phenotypic subpopulations were used as the training group to predict the oil content of the remaining subpopulation, the prediction accuracy were -0.13, 0.28, 0.37 and 0.18, respectively (**Table S2**). To confirm the genetic relationship of each subpopulation, PCA was conducted for each population using genotypic data, germplasms in Ppop1 and Ppop2 as well as, Ppop3 and Ppop4 tend to cluster together (**Figures S1**, **S2**). The prediction accuracy within the subpopulations was higher than that between the subpopulations, which means that the higher the genetic similarity between the training population and the prediction population, the higher the prediction accuracy. Exceptions occur when using Ppop 2 as the training population to predict the protein performance in Ppop 1, Ppop 2, Ppop 3 and Ppop 4, the highest prediction accuracy was not Ppop 2-Ppop 2. This situation also occurs when using Ppop 3 as the training population to predict the protein performance in Ppop 1, Ppop 2, Ppop 3 and Ppop 4. (**Figures 2D, E**).

### Effect of population structure on prediction accuracy

**Figure 3:**
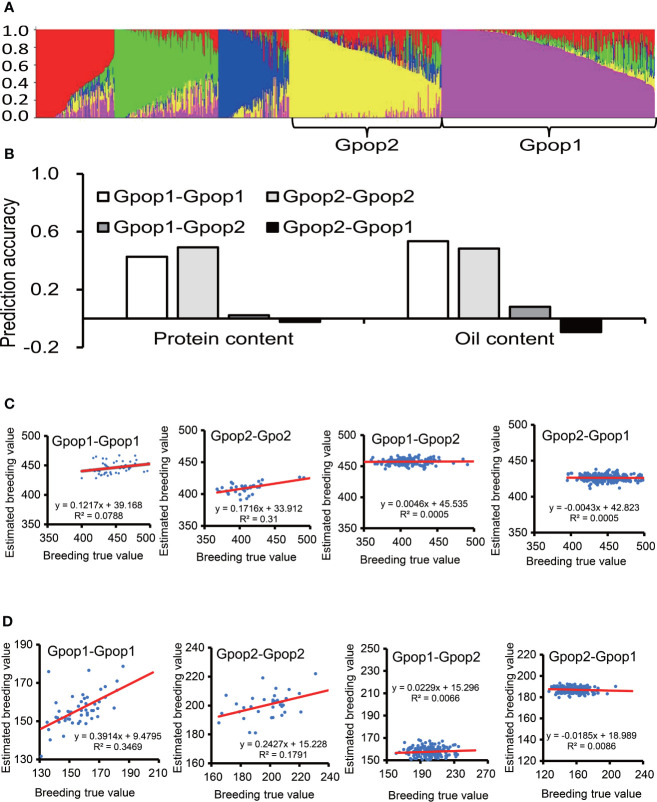
The effect of genetic similarity between the training population and prediction population. The prediction accuracies of RR-BLUP for protein and oil content among soybean germplasms grown at Urbana, IL in 2001 and 2002 **(B)**, when the training population and prediction population were selected according to genetic structure **(A)**. **(C)** Scatter plot of protein content, abscissa for breeding true value, ordinate for estimated breeding value, the red line is the trend line. **(D)** Scatter plot of oil content.

The STRUCTURE analysis did not produce a clear “plateau” as Ln P(D) increased gradually with the number of K from 2 to 6 for all the panels. The highest value of ΔK for the Max_IL_0102 panel was at K = 5 (**Figure 3A**). Hence, five subpopulations were inferred (**Table S2**). The numbers of accessions in each subpopulation were 259, 174, 108, 86 and 79. Due to the relatively large population size of subpopulations 1 and 2, they were used for subsequent analyses. The subpopulation 1 and 2 was also verified in PCA (**Figure S3**). For protein content, the prediction accuracy of samples in Gpop 1 as both training set and test set (Gpop 1- Gpop 1) was 0.43; the prediction accuracies of Gpop 2-Gpop 2, Gpop 1-Gpop 2 and Gpop 2-Gpop 1 were 0.49, 0.02 and -0.02, respectively. For oil content, the prediction accuracies of Gpop 1-Gpop 1, Gpop 2-Gpop 2, Gpop 1-Gpop 2 and Gpop 2-Gpop 1 were 0.53, 0.48, 0.08 and -0.09, respectively. When Gpop1,2,3,4,5 were combined as training population to predict the performance of the remaining population, the prediction accuracies for protein content were 0.13, 0.29, 0.18, 0.48, and 0.36, respectively, and for oil content, were 0.28, 0.41, 0.16, 0.39 and 0.43, respectively. The intragroup prediction accuracies were higher than the intergroup prediction accuracies (**Figure 3B**). When the prediction accuracy was positive, the real breeding value was positively correlated with the estimated breeding value. When the prediction accuracy was negative, the real breeding value had no correlation with the estimated breeding value (**Figures 3C, D**).

### Effect of marker selection strategies on prediction accuracy

**Figure 4:**
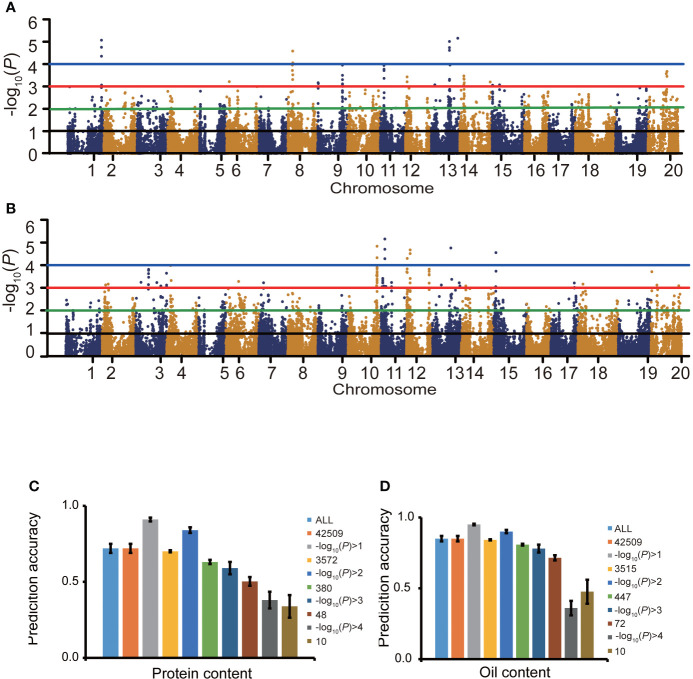
The effect of marker association with the trait on prediction accuracy. Manhattan plots for protein content **(A)** and oil content **(B)**. Effects of marker sampling strategies on cross-validated prediction accuracies of RRBLUP for protein content **(C)** and oil content **(D)** among 1,007 soybean germplasms grown at Urbana, IL in 2001 and 2002. Marker subsets were selected using random sampling **(C, D)** and an -log_10_(*P*) value-based sampling strategy **(A, B)**. The number of markers for random selection was the same as the number of markers selected based on the -log_10_(*P*) value.

For protein content, at the threshold of -log_10_(*P*)>0, 1, 2, 3 and 4, the number of SNPs related to protein content were 35,517, 3,572, 380, 48 and 10, respectively (**Figures 4A, C**), as identified by the GWAS. When -log_10_(*P*)>0, 1, 2, 3 and 4, the protein content prediction accuracy were 0.72, 0.91, 0.84, 0.59 and 0.38, respectively. When -log_10_(*P*)>1, the highest prediction accuracy was obtained (**Figure 4C**). The prediction accuracy decreased with fewer SNP markers (**Figure 4C**). For oil content, when -log_10_(*P*)>0, 1, 2, 3 and 4, the number of SNPs associated with oil content was 35,517, 3,515, 447, 72 and 10, respectively (**Figures 4B, D**). When -log_10_(*P*)>0, 1, 2, 3 and 4, the oil content prediction accuracy were 0.85, 0.95, 0.90, 0.78 and 0.36, respectively, and the prediction accuracy was the highest when -log_10_(*P*)>1. The prediction accuracy decreased with decreasing SNP numbers (**Figure 4D**). The prediction accuracies based on associated markers were generally higher than that based on random markers (**Figures 4C, D**).

## Discussion

With the development of sequencing technology, the speed of generating genotypic data has increased significantly. The acquisition of a large number of genotypic data enables the phenotypic data of the corresponding materials collected in previous years to be used for genomic selection.

The size of the training population and the predicted population affects prediction accuracy. Previous studies have shown that the accuracy of prediction increases with population size, when the population genetic diversity is high, a larger proportion of the training population is required (Piepho, 2009; Heffner et al., 2011). In this study, with the size increase in the training group, the prediction accuracy also slightly increased.

The transferability of the GS models across populations was assessed by estimating the prediction accuracies between pairwise populations when one population was used as a training set to predict performance in other populations. Guo et al. (2020) established training sets based on the phenotypic variation of the target trait. Four scenarios were simulated and compared in each of the four populations. The prediction accuracies were low across all pairwise populations, but high when the prediction population and training population were with similar phenotypic variation or the range of phenotypic variation of accessions in the training population covered the phenotypic value of accessions in the prediction population. The results indicated that the phenotypic similarity between training population and prediction population had the greatest impact on the prediction accuracy (**Table S1**). Developing a training set with broad phenotypic variation could improve the prediction accuracy.

Phenotype was related to genotype, when selecting a training population for GS, it was necessary to consider its genetic relationship with the prediction population. Prediction accuracy between populations with close genetic relationship was higher than that of groups with distant relationship. In natural populations, the population structure and genetic distance between training and prediction populations can affect the accuracy of marker effect estimation, which will affect the prediction performance of GS (Duangjit et al., 2016; Habyarimana, 2016). Esfandyari et al. (2015) reduced the impact of population structure on prediction accuracy by creating a training population with a close genetic relationship to the breeding population and a breeding population with a close genetic relationship to the training population. In this experiment, the prediction accuracy was higher within groups than between groups, confirming the influence of genetic relationships and population structure on the prediction accuracy. The effect of population structure on prediction accuracy is mainly due to the large difference in allele frequency between different subgroups in natural population, which affects the accuracy of marker effect estimation, such differences are difficult to evaluate and capture by statistical models (Liu et al., 2018; Rio et al., 2019). Considering the influence of the genetic similarity between the prediction population and the training population on the prediction accuracy, to improve the prediction accuracy, materials with genetic backgrounds similar to those of the prediction population should be selected to form the training population.

The number of markers is a key factor for the successful integration of GS into plant breeding programs. A large number of markers can capture most of the linkage information between QTLs and SNPs (Meuwissen et al., 2001; Solberg et al., 2008). Ma et al. (2016) discussed the relationship between prediction accuracy and the number of markers. The results showed that a decrease in marker density led to a slight decrease in prediction accuracy. In this experiment, when -log_10_(*P*)>1, a total of 3572 markers associated with protein content and 3515 SNP markers associated with oil content were identified, and using these markers in the GS achieved similar effects as using all SNPs. When SNPs of -log_10_(*P*)>2 for protein and -log_10_(*P*)>3 for oil content were used in GS, high prediction accuracies were also obtained for the traits. Although prediction accuracy increased with SNP number, inclusion of markers associated with targeted traits reduces costs while maintaining prediction accuracy.

## Conclusion

A training population with an appropriate population size, similar phenotypic value and close genetic relationship to the prediction population and the inclusion of markers significantly associated with the targeted traits increased prediction accuracy. The research results showed that GS is a potential powerful method for improving protein and oil content in soybean. The information from this study will help to optimize methods of applying GS to improve breeding efficiency.

## Data availability statement

Publicly available datasets were analyzed in this study. This data can be found here: https://www.soybase.org/dlpages/index.php and https://bigd.big.ac.cn/gvm/getProjectDetail?Project=GVM000445.

## Author contributions

BS, data curation and writing-original draft. RG, visualization of the work. ZL, project administration. XS and QY, resources. JYS, data curation. MZ and CY, supervision. SZ, JZ, JH, JPZ, and JHS, validation. QJS, resources, writing-reviewing, and editing. LY, conceptualization and methodology. All authors contributed to the article and approved the submitted version.

## References

[B1] Babu S. R. Meena K. Dudwal R. (2016). Population dynamics of major defoliators (semiloopers and tobacco caterpillar) in soybean crop. Legume Res. 40, 183–186. doi: 10.18805/lr.v0iof.4483

[B2] Beavis W. D. (1998). “QTL analysis: power, precision and accuracy,” in Molecular dissection of complex traits. Ed. Paterson A. (New York, NY: CRC Press), 145–162.

[B3] Bradbury P. J. Zhang Z. Kroon D. E. Casstevens T. M. Ramdoss Y. Buckler E. S. (2007). TASSEL: software for association mapping of complex traits in diverse samples. Bioinformatics 23, 2633–2635. doi: 10.1093/bioinformatics/btm308 17586829

[B4] Budhlakoti N. Kushwaha A. K. Rai A. Chaturvedi K. K. Kumar A. Pradhan A. K.. (2022). Genomic selection: A tool for accelerating the efficiency of molecular breeding for development of climate-resilient crops. Front. Genet. 13. doi: 10.3389/fgene.2022.832153 PMC8864149 35222548

[B5] Calenge F. Legarra A. Beaumont C. (2011). Genomic selection for carrier-state resistance in chicken commercial lines. BMC Proc. 5, S24. doi: 10.1186/1753-6561-5-S4-S24 PMC3108219 21645304

[B6] Cleveland M. A. Hickey J. M. (2013). Practical implementation of cost-effective genomic selection in commercial pig breeding using imputation. J. Anim. Sci. 91, 3583–3592. doi: 10.2527/jas.2013-6270 23736050

[B7] Duangjit J. Causse M. Sauvage C. (2016). Efficiency of genomic selection for tomato fruit quality. Mol. Breed. 36, 29. doi: 10.1,007/s11032-016-0453-3

[B8] Duchemin S. I. Colombani C. Legarra A. Baloche G. Larroque H. Astruc J. M.. (2012). Genomic selection in the French lacaune dairy sheep breed. Dairy Sc. 95, 2723–2733. doi: 10.3168/jds.2011-4980 22541502

[B9] Duhnen A. Gras A. Teyssèdre S. Romestant M. Claustres B. Daydé J.. (2017). Genomic selection for yield and seed protein content in soybean: A study of breeding program data and assessment of prediction accuracy. Crop Sci. 57, 1325–1337. doi: 10.2135/cropsci2016.06.0496

[B10] Endelman J. B. (2011). Ridge regression and other kernels for genomic selection with r package rrBLUP. Plant Genome 4, 250–255. doi: 10.3835/plantgenome2011.08.0024

[B11] Esfandyari H. Sorensen A. C. Bijma P. (2015). A crossbred reference population can improve the response to genomic selection for crossed performance. Genet. Sel. Evol. 47, 76. doi: 10.1186/s12711-015-0155-z 26419430 PMC4587753

[B12] Evanno G. Regnaut S. Goudet J. (2005). Detecting the number of clusters of individuals using the software STRUCTURE: a simulation study. Mol. Ecol. 14, 2611–2620. doi: 10.1111/j.1365-294X.2005.02553.x 15969739

[B13] Guo R. Dhliwayo T. Mageto E. K. Palacios-Rojas N. Lee M. Yu D.. (2020). Genomic prediction of kernel zinc concentration in multiple maize populations using genotyping-by-Sequencing and repeat amplification sequencing markers. Front. Plant Sci. 11. doi: 10.3389/fpls.2020.00534 PMC7225839 32457778

[B14] Habyarimana E. (2016). Genomic prediction for yield improvement and safeguarding of genetic diversity in CIMMYT spring wheat (*Triticum aestivum* l.). Aust. J. Crop Sci. 10, 127–136.

[B15] Heffner E. L. Jannink J. L. Iwata H. Souza E. Sorrells M. E. (2011). Genomic selection accuracy for grain quality traits in biparental wheat populations. Crop Sci. 51, 2597–2606. doi: 10.2135/cropsci2011.05.0253

[B16] Helms T. C. Hurburgh C. R. Lussenden R. L. Whited D. A. (1990). Economic analysis of increased protein and decreased yield due to de-layed planting of soybean. J. Produc. Agric. 3, 367–371. doi: 10.2134/jpa1990.0367

[B17] Jarquin D. Specht J. Lorenz A. (2016). Prospects of genomic prediction in the USDA soybean germplasm collection: Historical data creates robust models for enhancing selection of accessions. G3: Genes Genomes Genet. 6, 2329–2341. doi: 10.1534/g3.116.031443 PMC4978888 27247288

[B18] Lee G. J. Boerma H. R. Villagarcia M. R. Zhou X. Li Z. Gibbs M. (2004). A major QTL conditioning salt tolerance in s-100 soybean and descendent cultivars. Theor. Appl. Genet. 109, 1610–1619. doi: 10.1,007/s00122-004-1783-9 15365627

[B19] Liu X. Wang H. Guo Z. Xu X. Liu J. Wang S.. (2018). Factors affecting genomic selection revealed by empirical evidence in maize. Crop J. 6, 6341–6352. doi: 10.1016/j.cj.2018.03.005

[B20] Ma Y. Reif J. C. Jiang Y. Wen Z. Wang D. Liu Z.. (2016). Potential of marker selection to increase prediction accuracy of genomic selection in soybean (*Glycine max* l.). Springer Open Choice 36, 113. doi: 10.1,007/s11032-016-0504-9 PMC4965486 27524935

[B21] Maher B. (2008). Personal genomes: The case of the missing heritability. Nat. News 6, 18–23. doi: 10.1038/456018a 18987709

[B22] Melchinger A. E. Utz H. F. Schön C. C. (1998). Quantitative trait locus (QTL) mapping using different testers and independent population samples in maize reveals low power of QTL detection and large bias in estimates of QTL effects. Genetics 149, 383–403. doi: 10.1093/genetics/149.1.383 9584111 PMC1460144

[B23] Meuwissen T. H. E. Hayes B. J. Goddard M. E. (2001). Prediction of total genetic value using genome-wide dense marker maps. Genetics 157, 1819–1829. doi: 10.1093/genetics/157.4.1819 11290733 PMC1461589

[B24] Moreau L. Charcosse T. A. Hospital F. Gallais A. (1998). Marker-assisted selection efficiency in populations of finite size. Genetics 148, 1353–1365. doi: 10.1093/genetics/148.3.1353 9539448 PMC1460046

[B25] Moreau L. Charcosset A. Gallais A. (2004). Experimental evaluation of several cycles of marker-assisted selection in maize. Euphytica 137, 111–118. doi: 10.1023/B:EUPH.0000040508.01402.21

[B26] Piepho H. P. (2009). Ridge regression and extensions for genome wide selection in maize. Crop Sci. 49, 1165–1176. doi: 10.2135/cropsci2008.10.0595

[B27] Pritchard J. K. Stephens M. Donnelly P. (2000). Inference of population structure using multilocus genotype data. Genetics 155, 945–959. doi: 10.1093/genetics/155.2.945 10835412 PMC1461096

[B28] Pryce J. E. Daetwyler H. D. (2011). Designing dairy cattle breeding schemes under genomic selection: a review of international research. Anim. Prod. Sci. 52, 107–114. doi: 10.1071/AN11098

[B29] Purcell S. Neale B. Todd-Brown K. Thomas L. Ferreira M. A. Bender D.. (2007). PLINK: A tool set for whole-genome association and population-based linkage analyses. Am. J. Hum. Genet. 81, 559–575. doi: 10.1086/519795 17701901 PMC1950838

[B30] Qin J. Shi A. Song J. Wang M. Cao H. (2019). Genome-wide association study and genomic selection of amino acid concentrations in soybean seeds. Front. Plant Sci. 10. doi: 10.3389/fpls.2019.01445 PMC6873630 31803203

[B31] Ravelombola W. Qin J. Shi A. Song Q. Yuan J. Wang F.. (2021). Genome-wide association study and genomic selection for yield and related traits in soybean. PloS One 16, e0255761. doi: 10.1371/journal.pone.0255761 34388193 PMC8362977

[B32] Rio S. Mary-Huard T. Moreau L. Charcosset A. (2019). Genomic selection efficiency and *a priori* estimation of accuracy in a structured dent maize panel. Theor. Appl. Genet. 132 (1), 81–96. doi: 10.1,007/s00122-018-3196-1 30288553

[B33] Rodrigues J. I. D. Arruda K. M. A. Cruz C. D. Piovesan N. D. de Barros E. G. Moreira M. A. (2014). Biometric analysis of protein and oil contents of soybean genotypes in different environments. Pesqui. Agropecu. Bras. 49, 475–482. doi: 10.1590/S0100-204X2014000600009

[B34] Shen C. Zhang L. Huang L. Zheng S. Guo N. Zhao N.. (2020). Genome-wide association analysis of soybean water-soluble proteins in Chinese. Soybean Sci. 39, 509–517. doi: 10.1038/s41598-017-04685-7

[B35] Solberg T. R. Sonesson A. K. Woolliams J. A. Meuwissen T. H. (2008). Genomic selection using different marker types and densities. J. Anim. Sci. 86, 2447–2454. doi: 10.2527/jas.2007-0010 18407980

[B36] Song Q. Hyten D. L. Jia G. Quigley C. V. Fickus E. W. Nelson R. L.. (2013). Development and evaluation of SoySNP50K, a high-density genotyping array for soybean. PloS One 8, e54985. doi: 10.1371/journal.pone.0054985 23372807 PMC3555945

[B37] Song Q. Hyten D. L. Jia G. Quigley C. V. Fickus E. W. Nelson R. L.. (2015). Fingerprinting soybean germplasm and its utility in genomic research. G3: Genes genomes Genet. 5, 1999–2006. doi: 10.1534/g3.115.019000 PMC4592982 26224783

[B38] Spindel J. Begum H. Akdemir D. Virk P. Collard B. Redona E.. (2015). Genomic selection and association mapping in rice (*Oryza sativa* l.): effect of trait genetic architecture, training population composition, marker number and statistical model on accuracy of rice genomic selection in elite, tropical rice breeding lines. PloS Genet. 11, e1004982. doi: 10.1371/journal.pgen.1004982 25689273 PMC4334555

[B39] Xavier A. Muir W. M. Rainey K. M. (2016). Assessing predictive properties of genome-wide selection in soybeans. G3 (Bethesda) 6, 2611–2616. doi: 10.1534/g3.116.032268 27317786 PMC4978914

[B40] Xu Y. Ma K. Zhao Y. Wang X. Zhou K. Yu G.. (2021). Genomic selection: A breakthrough technology in rice breeding. Crop J. 9, 669–677. doi: 10.1016/j.cj.2021.03.008

[B41] Yan L. Di R. Wu C. Liu Q. Wei Y. Hou W.. (2019). Haplotype analysis of a major and stable QTL underlying soybean (*Glycine max*) seed oil content reveals footprint of artificial selection. Mol. Breed. 39 (34), 57. doi: 10.1,007/s11032-019-0951-1

[B42] Yu J. Pressoir G. Briggs W. H. Bi I. V. Yamasaki M. Doebley J. F.. (2006). A unified mixed-model method for association mapping that accounts for multiple levels of relatedness. Nat. Genet. 38, 203–208. doi: 10.1038/ng1702 16380716

[B43] Zhang Y. H. Liu M. F. He J. B. Wang Y. F. Xing G. N. Li Y.. (2015). Marker-assisted breeding for transgressive seed protein content in soybean [*Glycine max* (L.) merr.]. Theor. Appl. Genet. 128, 1061–1072. doi: 10.1,007/s00122-015-2490-4 25754423

[B44] Zhang J. Song Q. Cregan P. B. Jiang G. L. (2016). Genome-wide association study, genomic prediction and marker-assisted selection for seed weight in soybean (*Glycine max*). Theor. Appl. Genet. 129, 117–130. doi: 10.1,007/s00122-015-2614-x 26518570 PMC4703630

[B45] Zhu S. Walker D. R. Boerma H. R. Parrott W. A. (2006). Fine mapping of a major insect resistance QTL in soybean and its interaction with minor resistance QTLs. Crop Sci. 46, 1094–1099. doi: 10.2135/cropsci2005.06-0109

