# Supplementary material for: Genetic variation and marker−trait association affect the genomic selection prediction accuracy of soybean protein and oil content
Source: Front Plant Sci. 2022 Dec 13;13:1064623. doi: 10.3389/fpls.2022.1064623 (PMC9793221; doi:10.3389/fpls.2022.1064623)
Supplement: Supplementary file 1 [file DataSheet_1.docx]

Supplementary Material

**Supplementary Table 1.** The 1007 soybean germplasms were divided into 4 subpopulations according to their protein content and oil content (mg/g).

| **Trait** | **Subpop-**  **ulation** | **Min** | **Max** | **Average** | **SD** | **variance** | **Skewness** | **Kurtosis** | **Coefficient of variation (%)** |
| --- | --- | --- | --- | --- | --- | --- | --- | --- | --- |
| Protein | Ppop1 | 34.50 | 41.30 | 39.70 | 1.34 | 1.80 | -1.22 | 1.49 | 3.38 |
|  | Ppop2 | 41.40 | 43.20 | 42.30 | 0.51 | 0.26 | -0.40 | -1.11 | 1.19 |
|  | Ppop3 | 43.20 | 45.50 | 44.27 | 0.66 | 0.44 | 0.12 | -1.14 | 1.50 |
|  | Ppop4 | 45.60 | 52.90 | 47.34 | 1.44 | 2.07 | 1.08 | 0.83 | 3.04 |
| Oil | Ppop1 | 8.20 | 15.80 | 14.58 | 1.04 | 1.09 | -2.34 | 8.64 | 7.15 |
|  | Ppop2 | 15.80 | 17.20 | 16.47 | 0.42 | 0.18 | -0.89 | -1.27 | 2.57 |
|  | Ppop3 | 17.20 | 18.90 | 18.05 | 0.51 | 0.26 | -0.97 | -1.23 | 2.81 |
|  | Ppop4 | 18.90 | 24.00 | 20.52 | 1.12 | 1.26 | 0.71 | -0.05 | 5.48 |

**Supplementary Table 2.** The 1007 soybean germplasms were divided into 5 subpopulations based on population structure analysis.

| **Subpopulation** | **The number of soybean germplasm resources** | **Protein content (mg/g)** | **Oil content (mg/g)** |
| --- | --- | --- | --- |
| subpopulation 1 | 259 | 395-529 | 127-207 |
| subpopulation 2 | 174 | 345-491 | 161-235 |
| subpopulation 3 | 108 | 394-514 | 105-203 |
| subpopulation 4 | 86 | 375-493 | 130-215 |
| subpopulation 5 | 79 | 352-476 | 153-240 |

**Supplementary Table 3.** The prediction accuracy of different phenotypic subpopulation.

| **Trait** | **Ppop234-Ppop1** | **Ppop134-Ppop2** | **Ppop124-Ppop3** | **Ppop123-Ppop4** |
| --- | --- | --- | --- | --- |
| Protein content | 0.19 | 0.22 | 0.24 | 0.02 |
| Oil content | -0.13 | 0.28 | 0.37 | 0.18 |

**Supplementary Table 4.** The prediction accuracy of different genotypic subpopulation.

| **Trait** | **Gpop2345-Gpop1** | **Gpop1345-Gpop2** | **Gpop1245-Gpop3** | **Gpop1235-Gpop4** | **Gpop1234-Gpop5** |
| --- | --- | --- | --- | --- | --- |
| Protein content | 0.13 | 0.29 | 0.18 | 0.48 | 0.36 |
| Oil content | 0.28 | 0.41 | 0.16 | 0.39 | 0.43 |

**Supplementary Table 5.** The corresponding relationship between maturity group and geographical source.

| **MG** | **Country** | | | | | | | |
| --- | --- | --- | --- | --- | --- | --- | --- | --- |
| 0 | China |  |  |  | South Korea |  |  |  |
| I | China | Japan |  |  | South Korea |  | United States | Vietnam |
| II | China | Japan |  |  | South Korea |  | United States |  |
| III | China |  | North Korea | Russia | South Korea | Ukraine | United States |  |
| IV | China |  | North Korea |  |  |  | United States |  |
| V |  |  |  |  |  |  | United States |  |

**Supplementary Table 6.** The corresponding relationship between maturity group and phenotypic population.

| **Trait** | **Ppop** | **MG** |
| --- | --- | --- |
| Protein content | 1 | I, II, III, IV, V |
|  | 2 | 0, I, II, III, IV, V |
|  | 3 | 0, I, II, III, IV, V |
|  | 4 | 0, I, II, III, IV |
| Oil content | 1 | 0, I, II, III, IV, V |
|  | 2 | 0, I, II, III, IV |
|  | 3 | I, II, III, IV, V |
|  | 4 | 0, I, II, III, IV, V |

**Supplementary Table 7.** The PI of germplasms used.

| ID | | | | | | | | |
| --- | --- | --- | --- | --- | --- | --- | --- | --- |
| PI424005 | PI587967 | PI594156 | PI594608A | PI597446 | PI603397 | PI603494 | PI603698I | PI614155 |
| PI468907 | PI587980A | PI594158 | PI594608B | PI597464 | PI603398A | PI603495A | PI603704A | PI614806 |
| PI468919 | PI587980C | PI594160 | PI594609 | PI597474 | PI603398B | PI603496A | PI603705A | PI614807 |
| PI483459 | PI587981 | PI594164 | PI594611 | PI597475A | PI603399 | PI603496B | PI603706A | PI614808 |
| PI504481 | PI587982A | PI594166 | PI594612 | PI597475B | PI603400 | PI603497 | PI603707 | PI614832 |
| PI504482 | PI587982B | PI594167 | PI594614A | PI597477 | PI603401 | PI603498A | PI603715 | PI614833 |
| PI504484 | PI587983B | PI594170A | PI594614B | PI597478A | PI603402 | PI603501 | PI603716 | PI616498 |
| PI504485 | PI587987A | PI594178 | PI594615 | PI597478B | PI603403 | PI603502A | PI603717 |  |
| PI504486 | PI587989B | PI594198 | PI594618A | PI597479 | PI603404 | PI603502B | PI603718A |  |
| PI504487 | PI587991 | PI594200 | PI594618B | PI597480A | PI603405A | PI603502C | PI603718B |  |
| PI504490 | PI587999A | PI594208 | PI594618C | PI597480B | PI603405B | PI603502D | PI603719A |  |
| PI504493 | PI587999B | PI594227A | PI594618D | PI597481 | PI603406 | PI603504 | PI603719B |  |
| PI504494 | PI587999C | PI594227B | PI594619 | PI597482 | PI603407 | PI603505 | PI603719C |  |
| PI504500 | PI587999D | PI594233A | PI594620 | PI597483 | PI603408 | PI603511A | PI603721 |  |
| PI504501 | PI588008A | PI594233B | PI594625 | PI597484 | PI603409 | PI603511B | PI603723 |  |
| PI504503 | PI588008C | PI594235 | PI594626 | PI597485 | PI603410 | PI603515 | PI603724C |  |
| PI504508 | PI588016 | PI594247 | PI594632 | PI597486 | PI603411 | PI603526 | PI603725 |  |
| PI504509 | PI588026A | PI594250 | PI594633 | PI597487 | PI603412A | PI603527A | PI603726 |  |
| PI506420 | PI588026B | PI594252A | PI594635B | PI598124 | PI603412B | PI603531A | PI603727 |  |
| PI506590E | PI588026C | PI594252B | PI594637 | PI598222 | PI603413 | PI603531B | PI603728 |  |
| PI506838B | PI588028 | PI594255 | PI594638A | PI599299 | PI603415 | PI603533 | PI603729 |  |
| PI507174 | PI588033A | PI594268A | PI594638B | PI599300 | PI603416 | PI603541B | PI603735A |  |
| PI507196 | PI588033B | PI594268B | PI594644A | PI599509 | PI603417 | PI603542 | PI603735B |  |
| PI507268 | PI588033C | PI594280A | PI594644B | PI602059 | PI603418A | PI603543A | PI603738 |  |
| PI534647 | PI588033D | PI594280B | PI594646 | PI602060 | PI603418B | PI603543B | PI603742A |  |
| PI534648 | PI588050A | PI594280C | PI594649 | PI602490 | PI603418C | PI603543C | PI603742B |  |
| PI542042 | PI588052A | PI594280D | PI594650B | PI602491 | PI603418D | PI603544A | PI603742D |  |
| PI543794 | PI590931 | PI594280E | PI594651 | PI602492 | PI603419A | PI603545A | PI603743A |  |
| PI546375 | PI590932 | PI594282 | PI594670A | PI602497A | PI603419B | PI603545B | PI603743B |  |
| PI549045A | PI592389 | PI594283 | PI594670B | PI602499 | PI603419C | PI603546A | PI603744 |  |
| PI559932 | PI592524 | PI594286 | PI594682A | PI602500A | PI603420 | PI603546B | PI603745 |  |
| PI561233B | PI592899 | PI594288 | PI594685A | PI602500B | PI603421B | PI603547 | PI603746 |  |
| PI561233C | PI592901 | PI594289 | PI594685B | PI602501 | PI603422A | PI603548A | PI603747 |  |
| PI564524 | PI592907A | PI594295 | PI594687 | PI602502A | PI603422B | PI603549 | PI603750A |  |
| PI567344A | PI592907B | PI594296 | PI594689 | PI602502B | PI603423A | PI603550 | PI603750B |  |
| PI572239 | PI592907C | PI594301 | PI594690A | PI602593 | PI603423B | PI603551A | PI603751A |  |
| PI573008 | PI592907D | PI594304A | PI594691 | PI602993 | PI603424B | PI603551B | PI603751B |  |
| PI576440 | PI592911B | PI594304B | PI594692 | PI603148 | PI603424C | PI603551C | PI603752 |  |
| PI577798 | PI592912A | PI594314 | PI594693A | PI603155 | PI603424D | PI603553 | PI603753B |  |
| PI578057 | PI592912B | PI594393 | PI594693B | PI603156 | PI603425 | PI603554A | PI603755A |  |
| PI578363 | PI592913 | PI594398A | PI594695 | PI603157 | PI603426G | PI603554B | PI603755B |  |
| PI578364 | PI592921 | PI594398B | PI594696B | PI603158 | PI603427A | PI603555 | PI603755C |  |
| PI578365 | PI592926 | PI594399A | PI594708A | PI603159 | PI603427B | PI603556 | PI603755D |  |
| PI578367 | PI592927 | PI594399C | PI594710 | PI603160 | PI603427C | PI603558 | PI603755E |  |
| PI578368 | PI592928 | PI594401A | PI594712 | PI603162 | PI603428A | PI603559 | PI603756 |  |
| PI578369 | PI592929 | PI594401B | PI594713 | PI603165A | PI603428B | PI603560 | PI603757A |  |
| PI578370 | PI592930 | PI594401C | PI594714 | PI603165B | PI603428C | PI603561 | PI603757C |  |
| PI578374 | PI592932 | PI594401D | PI594777 | PI603166 | PI603428D | PI603562B | PI603758A |  |
| PI578375B | PI592933 | PI594401E | PI594778 | PI603167 | PI603429C | PI603563A | PI603758B |  |
| PI578376 | PI592934 | PI594402 | PI594779 | PI603168 | PI603429D | PI603563B | PI603758C |  |
| PI578378 | PI592935 | PI594403 | PI594780 | PI603171 | PI603430A | PI603564A | PI603759A |  |
| PI578380A | PI592936 | PI594404 | PI594790A | PI603172 | PI603430B | PI603564C | PI603760 |  |
| PI578380B | PI592937 | PI594405 | PI594792A | PI603174A | PI603431 | PI603567A | PI603764B |  |
| PI578382 | PI592938 | PI594406 | PI594795 | PI603174B | PI603432C | PI603567B | PI603777 |  |
| PI578383 | PI592939 | PI594407 | PI594798 | PI603175 | PI603433B | PI603568 | PI603909A |  |
| PI578384 | PI592940 | PI594408 | PI594799A | PI603176A | PI603434 | PI603569A | PI603909B |  |
| PI578388B | PI592941 | PI594409A | PI594799B | PI603290 | PI603436A | PI603569B | PI603909C |  |
| PI578390 | PI592942 | PI594409B | PI594800 | PI603293A | PI603436B | PI603570A | PI603910A |  |
| PI578392A | PI592944 | PI594410 | PI594801B | PI603298 | PI603438A | PI603570B | PI603910B |  |
| PI578399 | PI592945 | PI594411 | PI594802A | PI603302 | PI603438B | PI603570C | PI603911B |  |
| PI578401A | PI592946 | PI594412 | PI594804 | PI603303 | PI603438C | PI603571B | PI603911C |  |
| PI578401B | PI592947 | PI594413 | PI594807A | PI603304 | PI603438D | PI603571C | PI603912 |  |
| PI578401C | PI592948 | PI594415A | PI594807B | PI603305 | PI603438E | PI603574 | PI603913A |  |
| PI578401D | PI592949 | PI594419 | PI594817 | PI603306 | PI603441 | PI603576A | PI603913B |  |
| PI578402 | PI592950 | PI594420 | PI594818 | PI603307 | PI603442 | PI603579 | PI603913C |  |
| PI578405 | PI592951 | PI594430A | PI594819 | PI603308A | PI603443A | PI603582 | PI603914 |  |
| PI578406 | PI592952 | PI594430B | PI594821A | PI603308B | PI603443B | PI603583 | PI603915C |  |
| PI578407 | PI592953 | PI594438 | PI594821B | PI603309 | PI603443C | PI603585A | PI603915D |  |
| PI578408 | PI592954 | PI594439B | PI594823 | PI603310 | PI603444A | PI603585B | PI603915E |  |
| PI578409A | PI592956A | PI594440 | PI594845 | PI603312 | PI603444B | PI603586 | PI603916 |  |
| PI578409B | PI592956B | PI594441 | PI594853 | PI603321 | PI603444C | PI603587A | PI603917 |  |
| PI578410 | PI592956C | PI594442A | PI594861 | PI603323 | PI603445A | PI603587B | PI604100 |  |
| PI578411 | PI592957 | PI594442B | PI594871 | PI603324B | PI603445B | PI603587C | PI606748 |  |
| PI578412 | PI592958 | PI594443 | PI594872 | PI603327 | PI603446 | PI603589 | PI606749 |  |
| PI578413 | PI592959 | PI594444A | PI594873 | PI603329 | PI603447 | PI603592 | PI607385 |  |
| PI578414 | PI592960 | PI594444B | PI594878 | PI603330 | PI603448 | PI603593 | PI608438 |  |
| PI578416 | PI592967 | PI594445 | PI594882A | PI603331 | PI603449 | PI603594 | PI608726 |  |
| PI578417B | PI592968 | PI594446 | PI594885A | PI603332 | PI603451B | PI603596 | PI610670 |  |
| PI578418 | PI592969 | PI594448A | PI594886 | PI603335B | PI603452 | PI603597 | PI610671 |  |
| PI578419A | PI592970 | PI594451 | PI594889 | PI603336 | PI603453 | PI603598A | PI611112 |  |
| PI578419B | PI592971 | PI594452 | PI594899A | PI603337A | PI603454 | PI603599B | PI612594 |  |
| PI578420 | PI592972 | PI594453 | PI594899B | PI603338 | PI603455A | PI603612 | PI612612A |  |
| PI578421 | PI592973 | PI594454A | PI594900A | PI603339B | PI603455B | PI603614A | PI612612B |  |
| PI578425 | PI592974 | PI594454B | PI594900B | PI603340 | PI603456 | PI603620 | PI612616 |  |
| PI578431 | PI592977 | PI594456A | PI594902 | PI603341 | PI603457A | PI603623 | PI612617A |  |
| PI578432B | PI592978 | PI594456B | PI595081 | PI603347 | PI603457B | PI603645 | PI612711B |  |
| PI578439 | PI592979 | PI594457A | PI595363 | PI603348A | PI603457C | PI603647 | PI612713A |  |
| PI578440 | PI592980 | PI594457B | PI595753 | PI603348B | PI603458A | PI603648 | PI612714B |  |
| PI578473A | PI592981 | PI594459 | PI595754 | PI603349 | PI603458B | PI603653 | PI612715 |  |
| PI578473B | PI593256 | PI594460 | PI595843 | PI603350 | PI603459 | PI603654 | PI612716 |  |
| PI578477A | PI593258 | PI594461A | PI595926 | PI603352 | PI603460 | PI603655 | PI612717 |  |
| PI578477B | PI593259 | PI594461B | PI596407 | PI603354 | PI603462 | PI603656 | PI612722 |  |
| PI578477C | PI593463 | PI594462 | PI597381 | PI603355 | PI603463 | PI603658 | PI612723 |  |
| PI578478A | PI593654 | PI594463A | PI597382 | PI603356 | PI603464 | PI603660 | PI612724 |  |
| PI578479 | PI593942 | PI594463B | PI597386 | PI603357 | PI603465B | PI603661A | PI612725 |  |
| PI578481 | PI593943 | PI594464 | PI597387 | PI603358A | PI603465D | PI603662A | PI612726 |  |
| PI578490 | PI593949A | PI594466 | PI597397A | PI603360 | PI603466A | PI603662B | PI612729 |  |
| PI578492 | PI593953 | PI594467 | PI597397B | PI603361 | PI603466B | PI603663 | PI612730 |  |
| PI578493 | PI593956A | PI594469A | PI597404 | PI603365 | PI603467 | PI603666 | PI612731 |  |
| PI578494A | PI593956B | PI594469B | PI597405B | PI603366 | PI603468 | PI603667A | PI612732 |  |
| PI578494B | PI593956C | PI594471A | PI597405C | PI603367 | PI603469 | PI603667B | PI612733 |  |
| PI578495 | PI593956D | PI594471B | PI597405D | PI603369 | PI603470 | PI603670 | PI612735 |  |
| PI578497A | PI593956E | PI594476 | PI597406 | PI603371 | PI603471 | PI603671 | PI612736 |  |
| PI578497B | PI593958 | PI594480A | PI597407A | PI603372 | PI603472A | PI603672A | PI612737 |  |
| PI578498A | PI593959 | PI594486A | PI597407B | PI603373 | PI603472B | PI603673A | PI612741 |  |
| PI578499A | PI593960 | PI594488 | PI597408 | PI603374 | PI603473 | PI603673C | PI612744 |  |
| PI578499B | PI593961 | PI594489 | PI597411A | PI603375 | PI603474 | PI603673D | PI612746 |  |
| PI578499C | PI593964 | PI594496 | PI597411B | PI603377 | PI603475 | PI603674 | PI612747 |  |
| PI578504 | PI593967 | PI594501A | PI597412 | PI603378A | PI603477B | PI603675 | PI612748 |  |
| PI578505 | PI593968 | PI594550 | PI597415 | PI603378B | PI603478 | PI603676 | PI612749 |  |
| PI583364 | PI593969 | PI594576 | PI597419 | PI603381A | PI603479 | PI603678A | PI612750 |  |
| PI583366 | PI593970 | PI594578 | PI597420 | PI603381B | PI603480 | PI603678B | PI612753B |  |
| PI583835 | PI593971 | PI594580 | PI597421 | PI603381C | PI603482 | PI603682 | PI612754 |  |
| PI583837 | PI593972 | PI594581 | PI597427A | PI603383 | PI603483 | PI603686 | PI612758B |  |
| PI584441 | PI593973 | PI594582 | PI597427B | PI603384 | PI603485 | PI603687A | PI612759D |  |
| PI584469 | PI593975 | PI594583 | PI597429 | PI603385 | PI603486 | PI603687B | PI612760 |  |
| PI584470 | PI593997 | PI594587 | PI597432 | PI603387 | PI603487A | PI603690 | PI612761B |  |
| PI586980 | PI594001 | PI594593 | PI597439 | PI603388 | PI603487B | PI603691 | PI612763 |  |
| PI586981 | PI594010 | PI594599 | PI597440B | PI603389 | PI603487C | PI603692 | PI612764 |  |
| PI587185 | PI594016 | PI594600 | PI597440C | PI603390A | PI603488 | PI603694A | PI612930 |  |
| PI587607A | PI594019 | PI594601 | PI597441 | PI603392 | PI603489 | PI603694B | PI612931 |  |
| PI587636 | PI594020 | PI594603A | PI597442 | PI603393 | PI603490 | PI603698A | PI612932 |  |
| PI587637 | PI594022 | PI594604 | PI597443 | PI603394 | PI603491 | PI603698B | PI614088 |  |
| PI587804 | PI594146 | PI594606 | PI597444 | PI603395 | PI603492 | PI603698D | PI614153 |  |
| PI587845 | PI594153 | PI594607 | PI597445 | PI603396 | PI603493 | PI603698E | PI614154 |  |


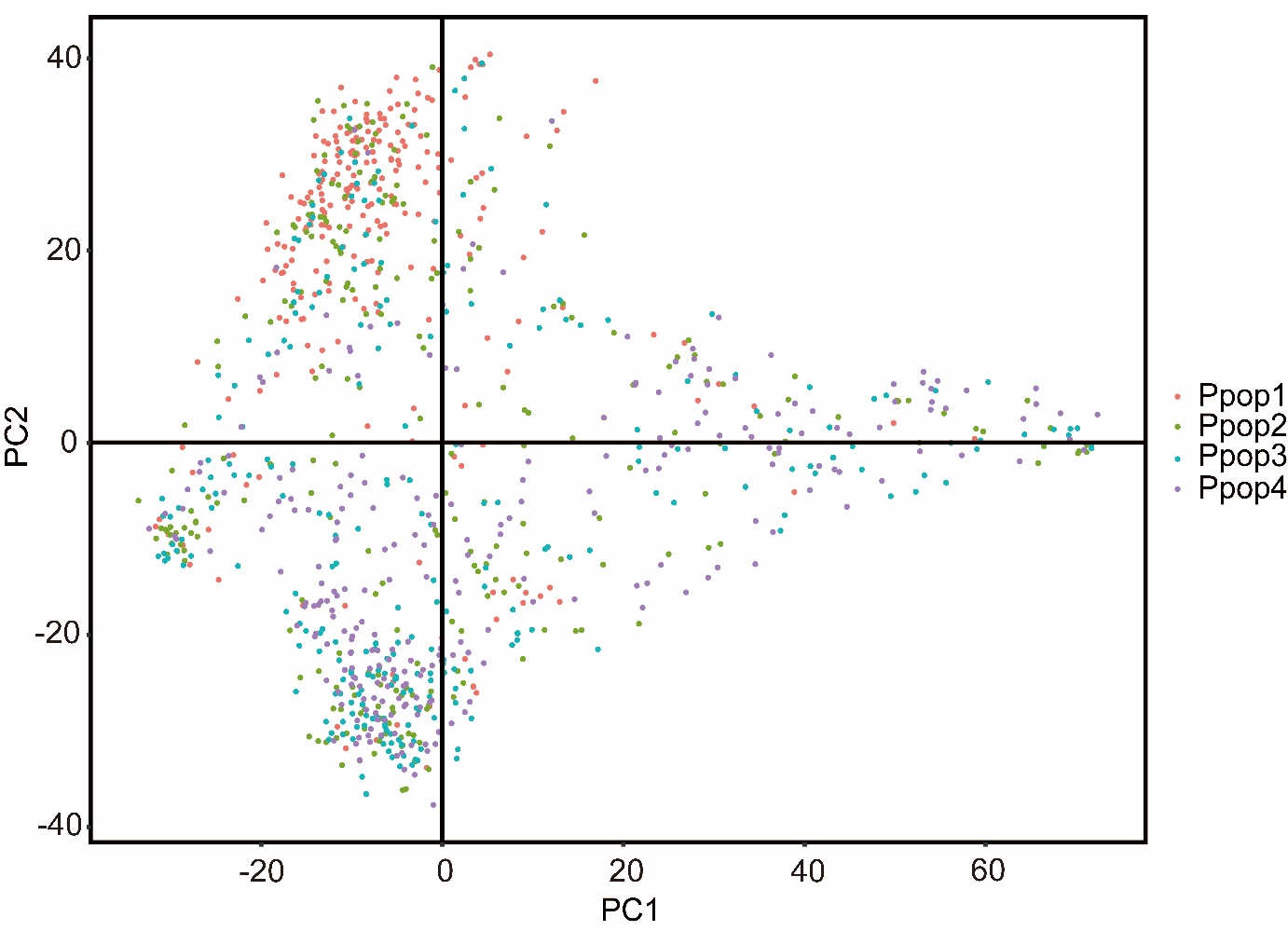


**Supplementary Figure 1.** **The PCA result of 4 subpopulations divided by protein content.** Each dot indicates a germplasm, “orange” indicates Ppop 1, “green” indicates Ppop 2, “blue” indicates Ppop 3 and “purple” indicates Ppop 4.


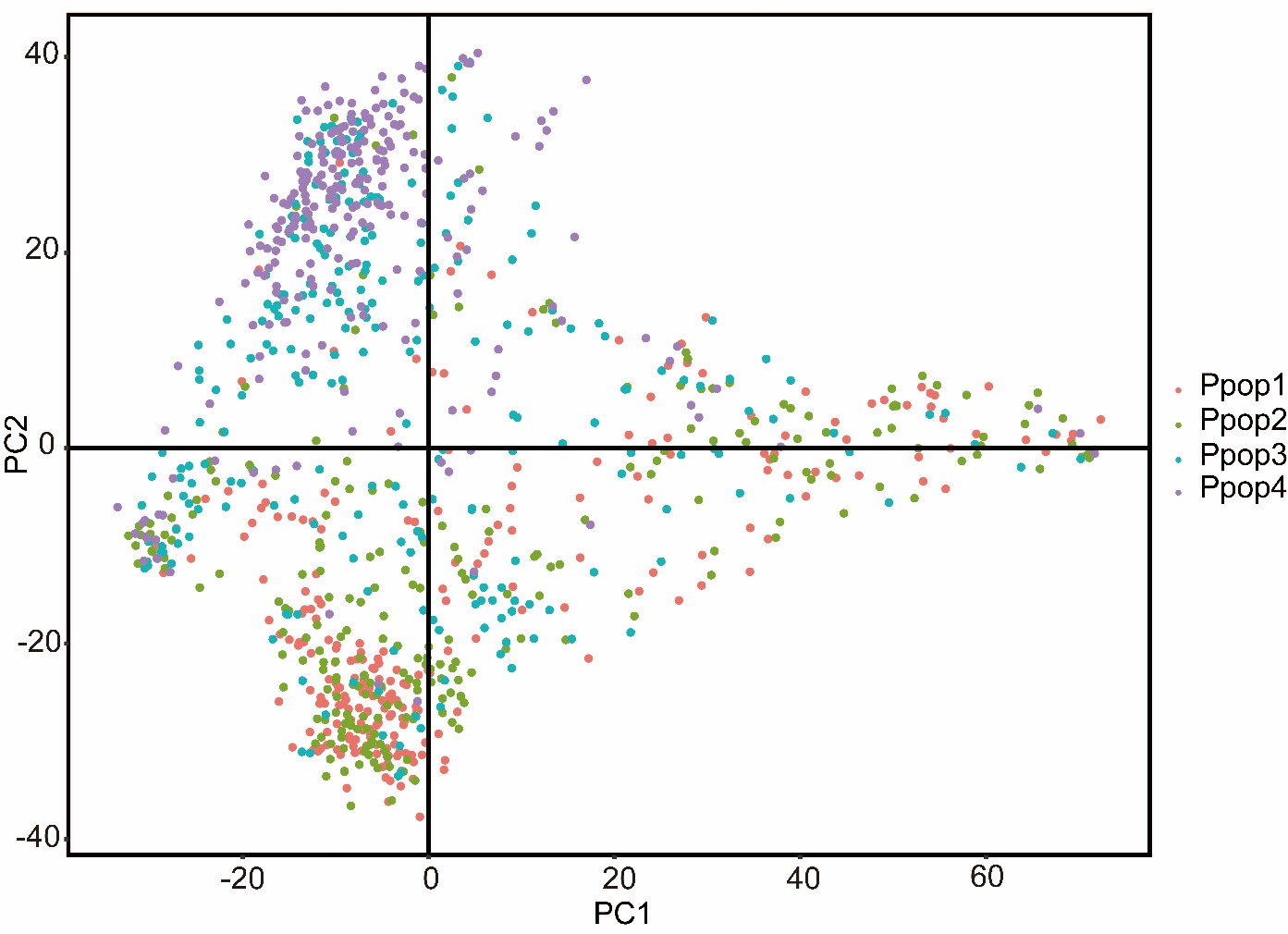


**Supplementary Figure 2. The PCA result of 4 subpopulations divided by oil content.** Each dot indicates a germplasm, “orange” indicates Ppop 1, “green” indicates Ppop 2, “blue” indicates Ppop 3 and “purple” indicates Ppop 4.


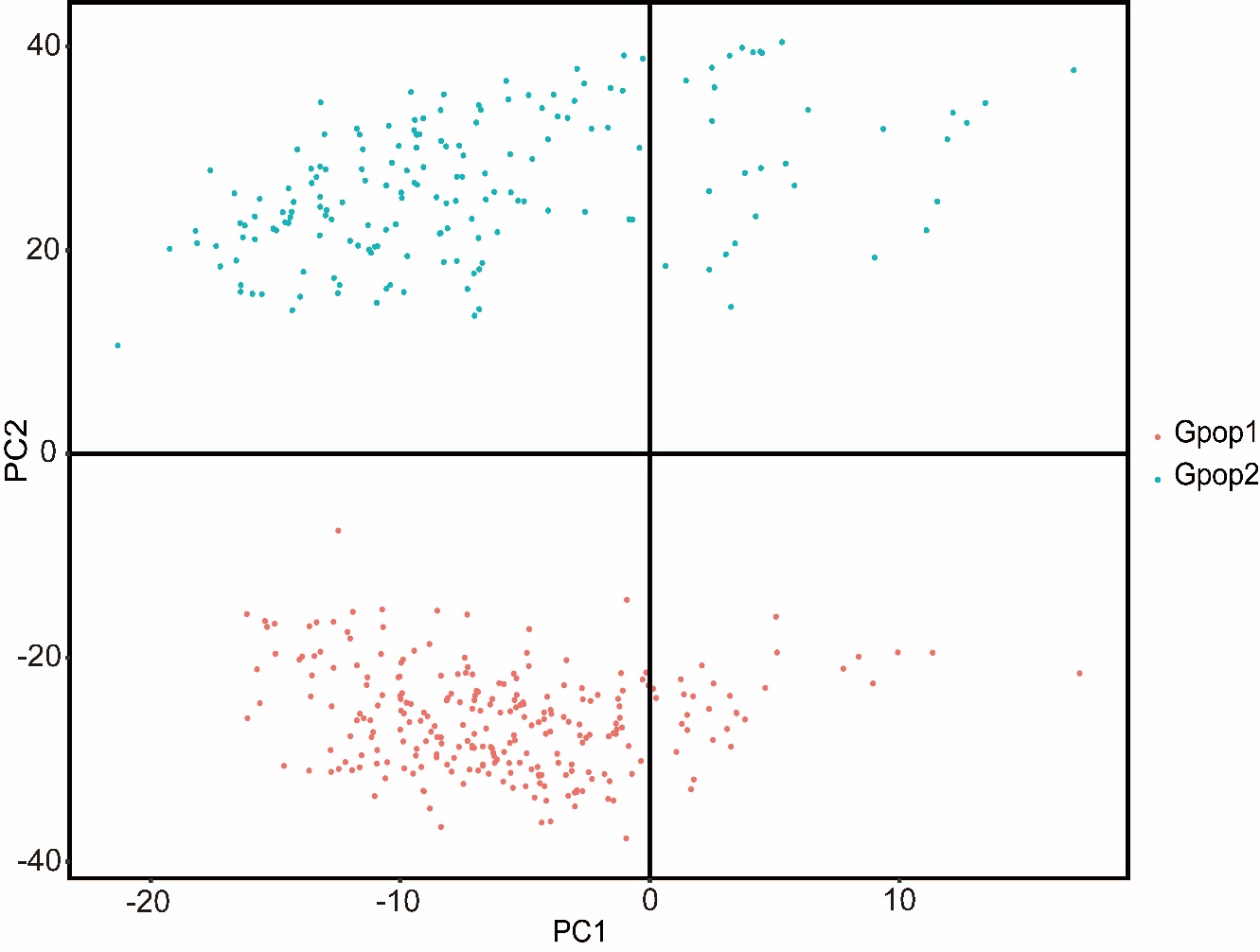


**Supplementary Figure 3. The PCA result of 2 subpopulations divided by genotypic data.** Each dot indicates a germplasm, “pink” indicates Gpop 1, “green” indicates Gpop 2.
